# Molecular dissection of a hyper-aggressive CBFB-MYH11/FLT3-ITD–positive acute myeloid leukemia

**DOI:** 10.1186/s12967-022-03486-5

**Published:** 2022-07-06

**Authors:** Gabriele Lo Iudice, Eleonora De Bellis, Arianna Savi, Luca Guarnera, Alice Massacci, Francesca De Nicola, Frauke Goeman, Tiziana Ottone, Mariadomenica Divona, Matteo Pallocca, Maurizio Fanciulli, Maria Teresa Voso, Gennaro Ciliberto

**Affiliations:** 1 SAFU Unit, IRCCS Regina Elena National Cancer Institute, Rome, Italy; 2 Department of Biomedicine and Prevention, Tor Vergata University, Rome, Italy; 3 Department of Onco-Hematology, Policlinico Tor Vergata, Rome, Italy; 4 Biostatistics, Bioinformatics and Clinical Trial Center, IRCCS Regina Elena National Cancer Institute, Rome, Italy; 5 Santa Lucia Foundation, IRCCS, Neuro-Oncohematology, Rome, Italy; 6 Laboratory of Advanced Diagnostics in Oncohematology, Hematology Department, Tor Vergata Hospital, Rome, Italy; 7 Scientific Direction, IRCCS Regina Elena National Cancer Institute, Rome, Italy

## Abstract

Acute Myeloid Leukaemia (AML) is a haematological malignancy showing a hypervariable landscape of clinical outcomes and phenotypic differences, explainable by heterogeneity at the cellular and molecular level. Among the most common genomic alterations, *CBFB-MYH11* rearrangement and *FLT3*-ITD gene mutations, have opposite clinical significance and are unfrequently associated. We present here a Molecular Case Report in which these two events co-exist an ultra-aggressive phenotype resulting in death in 4 days from hospital admittance. Somatic and germline Whole Exome Sequencing analysis was performed to uncover other putative driver mutations, de-novo genomic structural events or germline clusters increasing cancer insurgence. Only three mutations in *LTK*, *BCAS2* and *LGAS9* were found, unlikely causative of the exhibited phenotype, prompting to additional investigation of the rare *CBFB-MYH11/ FLT3-*ITD scenario.

**Supplementary Information:**

The online version contains supplementary material available at 10.1186/s12967-022-03486-5.

## Background

Acute Myeloid Leukaemia (AML) is a heterogeneous disease characterized by clonal expansion of undifferentiated myeloid precursors with variable phenotype and relevant molecular differences. This heterogeneity, coupled with the high relapse rate is responsible for the relatively low five-year overall survival (OS) for childhood AML, which lays at around 70%, compared to 90% for the more common acute lymphoblastic leukaemia [1, 2]. Indeed, over 30% of patients achieving complete remission (CR) after intensive chemotherapy develop relapses which are usually drug-resistant, with significant worsening of prognosis [1, 3].

Nowadays, the evolution of sequencing technologies allows for a genomic-based differential diagnosis for this form of cancer, with the identification of key mutations as new criteria to distinguish different forms of AML, granting more accurate prognostication [4], and the development of new, specific pharmacologic treatments [5]. Nonetheless, our understanding of the synergies of concurrent genomic alterations is still incomplete. Most patients are screened for the presence of known genomic anomalies only, without accounting for the role of co-occurrences in driving development of resistance, and epigenetic reprogramming of cancer clones is not well established [6].

In this article, we present the unusual case of early death in a child with a *FLT3*-ITD positive *CBFB-MYH11* rearranged AML, characterized by trio-filtered Whole Exome Sequencing (WES) analysis, and a comparison to available published data, to foster the identification of molecular determinants of disease aggressiveness in this case.

## Methods

All samples were equally treated for gDNA extraction from PBMCs using QIAamp DNA Mini Kit (Qiagen). The quantity and quality of the DNA was assessed by the Qubit and by NanoDrop Spectrophotometer, respectively. DNA exome libraries for sequencing were generated using 150 ng of DNA according to the TruSeq DNA Exome kit (Illumina). Quality of the libraries has been verified with the Bioanalyzer (High Sensitivity DNA Kit); library quantity has been determined with qPCR. The samples have then been sequenced in paired-end mode, sequencing from each side 76 bp with NextSeq 500 (Illumina).

Somatic and Germline variants were called via the Dragen Somatic/Germline pipelines available on the Illumina Basespace Cloud (vv. 3.6.3). VCF files were downloaded, normalized and decomposed via the VT package (Tan et al., 2015) and intersected via custom R scripting. A final manual curation was performed to validate called somatic SNPs. Copy Number Variations were called via the CNVkit package (Talevich et al. 2014) batch algorithm. CNV baseline was established with all the available in-house data sequenced (N = 8) with the same library preparation kit in order to smooth out technical biases. Only CNVs with significant p value (< 1e−10) and a strong effect on deletion (CN ≤ 1) or amplification (CN ≥ 4) were considered. Structural Variations were computed with Manta (Chen et al. 2016), excluding events flagged as imprecise and other events that were exceeding the insert size length according to 75 bp read length. One section of our results is partially based upon data generated by the Therapeutically Applicable Research to Generate Effective Treatments initiative, phs000218. The data used for this analysis are available at https://portal.gdc.cancer.gov/projects.

## Results

A fourteen-year-old female, with no previous history of diseases, was hospitalized after referral from the general practitioner, presenting worsening nausea and fever, and ocular hyposphagma. Upon reaching the hospital (day 1) she developed dyspnoea and peripheral blood (PB) tests showed 364 × 10^9^/L white blood cells (WBC, normal values: 4–12 × 10^9^/L), prompting AML diagnosis. Cytarabine was started as continuous infusion, 150 mg IV every twelve hours, followed by Idarubicin, 10 mg, and dexamethasone, 6 mg from day 2. On the same day, the patient was moved to the paediatric intensive care unit for respiratory failure, eventually needing intubation by the end of the day. The patient, now showing hepatosplenomegaly and peripheral bleeding manifestations, was awake until sedation by administration of midazolam. On day 3, patient conditions further worsened, and transfusion support was started, while WBC showed only a moderate decrease (215 × 10^9^/L). On day 4, after multiple organ failures (MOF), the patient died due to a mesencephalic haemorrhage.

Upon hospitalization, the patient was screened for genetic alterations commonly associated with AML, detecting both a *FLT3*-ITD mutation and *CBFB-MYH11* rearrangement. She was negative for *RUNX1/RUNX1T1*, *BCR-ABL1*, *MLL (KMT2A)* rearrangements, *FLT3*-TKD and *NPM1* mutations. Polymerase chain reaction (PCR) analysis of primary cells in accordance with European Leukemia Net (ELN) guidelines [7], confirmed the presence of a *FLT3*-ITD (Internal Tandem Duplication) with an Allelic Ratio (AR) of 0.15. Sequencing of the gene showed a 7 bp insertion in the juxta membrane domain of *FLT3*, associated with a 62 bp tandem duplication spanning to the TK Domain 1. Analysis of karyotype confirmed an inv(16). The association of *FLT3*-ITD mutations with inv(16) is a rare event in the AML landscape [8], and given the aggressive disease onset and the rapid fatal outcome, we decided to characterize in depth the genomic features of this AML case.

This study was approved by the Regina Elena Ethics Committee (CE 1353/20). Written informed consent was collected from the parents before blood sampling, on behalf of their child for the publication of any potentially identifiable data included in this article, and clinical data were collected from the patient chart and her parents.

**Fig. 1:**
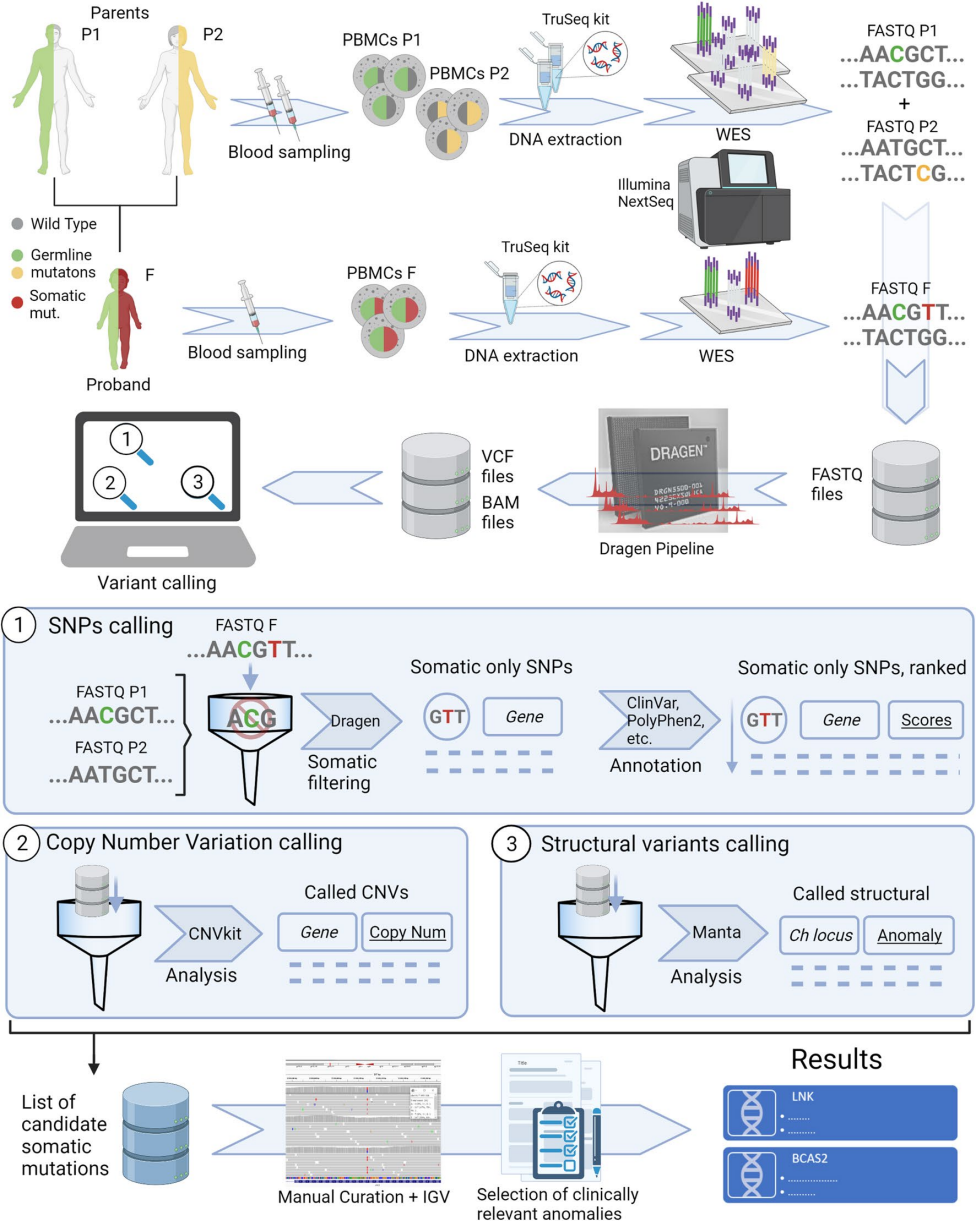
Workflow of the case molecular investigation. In order to apply a Trio analysis, peripheral blood samples from both the patient (proband, F) and the parents (P1, P2) were collected. After isolation, mononuclear cells were lysed, then DNA was extracted and purified to perform Whole Exome Sequencing. Resulting data has been analysed exploiting Illumina tools and other software, coupled with custom R scripts. This allowed the selection of relevant anomalies, which were then manually screened

Whole Exome Sequencing (WES) was performed on blast cells. In absence of a germline sample, PB Mononuclear Cells (PBMCs) samples were obtained from both parents to perform a family trio analysis of genetic variants, in order to achieve higher sensitivity and ease of data filtering compared to patient WES alone [9]. The methods are presented visually in Fig. 1 and expanded in the supplementary section. After filtering variants shared with the parents, only two potentially relevant Single Nucleotide Variants (SNVs) were identified, presented in Table 1.

**Table 1:** Description of the AML-only mutations filtered via trio Whole-Exome Sequencing. Public Database annotations are provided for each mutation along with a brief literature background

GENE	Mutation	Annotation	Functional impact	Reference
LTK	Missense SNV: Ch15: 41,803,754 p. P227L 680C > T VAF:0.57 COSM3749294, COSM3749295 rs55739813	SIFT:0 Polyphen:1	Leukocyte tyrosine kinase, often overexpressed in leukaemia, unknown ligand	[35]
BCAS2	Missense SNV Ch1: 115,118,313 p.C106Y 317G > A VAF:0.47	SIFT:0 Polyphen:0,98	Encodes pre-mRNA splicing factor SPF27, probable oncogenic role in breast cancer, possible role in haematopoiesis (known role in zebrafish)	[36]
LGALS9	CNV up (cn = 4) Ch17: 18,380,138–18,397,683 Depth: 537,146	N/A	TIM3 ligand, soluble cytokine. Promotes apoptotic pathways, confers immune avoidance, promotes hypoxia coping mechanisms; pathways are not currently known	[37]

Analysis of gene Copy Number Variations (CNVs) revealed a focal shallow deletion (CN = 1) on the *TUBB8B* gene, and two shallow larger events on chr4 and chr17. Other two large deletions were discarded since overlapping the validated inversion on chr16. When considering significant amplifications with a strong signal (CN ≥ 4), only one event emerged, presented in Table 1. A further analysis on other genomic structural events such as translocations, insertions, and inversions, was performed: of the four alterations detected, one was excluded via further manual filtering (imprecise breakpoints). The three remaining insertions on chr3, chr9 and chr20 were not considered since they exceeded the software length threshold for insert size.

We then assessed the prevalence of concurrent *FLT3*-ITD and *CBFB-MYH11* across several patient cohorts exploiting the cBioPortal [10]interface. The overall population of double-mutant is comprising of 5 patients (0.5%) in the TARGET study and 2 patients (1%) in the TCGA study, in agreement with previously published data [11–15]. Interestingly, none of the mutations described in Table 1 could be observed in the somatic landscape of the double-mutated population. Furthermore, when querying for mutational events (SNPs, Indels, and CNVs) on LTK, BCAS2, LGASL2, and TUBB8B, the amount of somatic point mutations was none on said patients, while only 2 Copy Number Amplifications in BCAS2 and 1 in LTK were found. Taken together, these results could point out to a molecular background that acts in a sinergistic manner with the *FLT3*-ITD + *CBFB-MYH11* combination.

We then sought to provide an overview of the prognostic value of all these variants on Overall Survival, while unfortunately such scarce numerosity of events prevents reaching statistical significance. Nonetheless, Overall Survival on all considered datasets suggests that these patients have worse prospects and a higher chance of relapse, especially in the paediatric population,

The median survival was 17 months on the 7 combined cases, against a median OS of 34.5 for FLT3-only, 59 for *CBFB-MYH11* only and 47 for none of these events (Additional file 1: Fig. S1A).

Finally, blast median count was 61.5, 79.5, 73, 70 on the 4 groups, confirming the double mutant scenario not be the sole hallmark of hyperleukocytosis (Additional file 1: Fig. S1B).

## Conclusions

To usher the era of precision medicine, a detailed understanding of the significance of genetic alterations is required. Indeed, not only their presence but also the interaction between the different genetic abnormalities, should be considered to explain the variability in response to treatment observed in patients.

The category of core binding factor (CBF) acute myeloid leukaemias (AMLs) includes AML with inv(16)(p13.1q22) and AML with t(8;21) (q22;q22,1), which translates into *CBFβ/MYH11* and *RUNX1/RUNX1T1* fusion genes, respectively [16]. These abnormalities have an incidence of 10–15% in adults and 20% in childhood de novo AMLs, and they are considered prognostically favourable [7, 16, 17]. The presence of CBF-AML chimeric transcripts causes disruption of the CBF complex with consequent block of differentiation of myeloid blasts [16]. However, several evidences suggest that the leukemogenesis process is more complex and could be supported by additional mutational events [18]. In 2002, Kelly et al. proposed a two-hit model for CBF leukaemia in which the proliferative advantage was conferred by a mutation in a gene coding for a tyrosine kinase [19]. The detection of an additional mutation in CBF-AMLs dates back to 2003, when Care et al. demonstrated that mutations of *c-Kit* or *FLT3* genes could be found in 40% of AML with *CBFβ/MYH11* cases [20]*.* Otherwise, subsequent studies reported the co-existence of a mutually exclusive mutational process for activating tyrosine kinases pathway in up to 70% of CBF-AML cases [21, 22].

*FLT3* mutations can be subdivided into internal tandem duplicates (ITD) that are in-frame duplications of variable size located within the juxatamembrane domain, and point mutations in the tyrosine kinase domain (TKD). *FLT3*-TKD mutations are observed in 11–13% of AMLs with *CBFβ/MYH11,* and they do not seem to affect prognosis [23–25], and in the paediatric population only anecdotal cases are reported [26]. *FLT3*-ITD mutations are even more rare in *CBFβ/MYH11* AMLs*,* with an incidence of 3 to 8%, and their prognostic role is negative. A mouse model study reported aggressive behaviour, low platelet counts and rapid peripheral dissemination of blasts [27]. Moreover, a retrospective collection of cases described poorer outcome of adult patients, as compared to those with CBF-AMLs without *FLT3*-ITD mutations. In literature, no data is available on *FLT3*-ITD-positive CBF-AMLs in paediatric population [28]. The presence of *FLT3*-ITD mutations confers to leukemic cells a proliferative and survival advantage by constitutive activation of downstream signalling events involving PI3K/AKT/mTOR, MEK/ERK and STAT5 pathways [29]. Consequently, these mutations correlate with hyperleukocytosis and poor prognosis [30–32].

Our patient presented at onset with all the biological features described above, including hyperleukocytosis, which is a known independent prognostic factor for inferior outcome, poor response to therapy and aggressive course of the disease [33]. It remains to be clarified whether a *FLT3*-ITD double mutation may have contributed to enhancing the unfavourable features known to be associated with a single mutation in this gene. Only another case of a 54-year-old patient with a de novo AML with *FLT3*-ITD double mutation is reported in the literature, with a particularly aggressive course and adverse outcome [34].

In conclusion, we reported the unique molecular profile of a paediatric patient affected by a *CBFβ/MYH11* AML, carrier of two *FLT3*-ITD co-mutations. Even though the genetic features would classify this AML as favourable risk, according to the 2017 ELN risk-stratification [7], the course of the disease was unusually rapid and fatal. This could be partly explained by the high leukocyte count at presentation. Further studies are needed to assess the clinical significance of a *FLT3*-ITD double mutations and clarify their prognostic features in CBF-AMLs.

## Supplementary Information

**Additional file 1: Figure S1A.** Kaplan–Meier curve showing Overall Survival among double mutant, inv16-only, FLT3-ITD only, and double negative cases in TCGA and TARGET studies. **B:** Blast count distribution in the 4 populations.

## Data Availability

List of annotated mutations are available upon request.

## Abbreviations

AML: Acute Myeloid Leukemia
SNVs: Single Nucleotide Variants
CNVs: Copy Number Variation
WES: Whole-Exome Sequencing
