# Supplementary figures and images for: Molecular dissection of a hyper-aggressive CBFB-MYH11/FLT3-ITD–positive acute myeloid leukemia
Source: J Transl Med. 2022 Jul 6;20:311. doi: 10.1186/s12967-022-03486-5 (PMC9258203; doi:10.1186/s12967-022-03486-5)

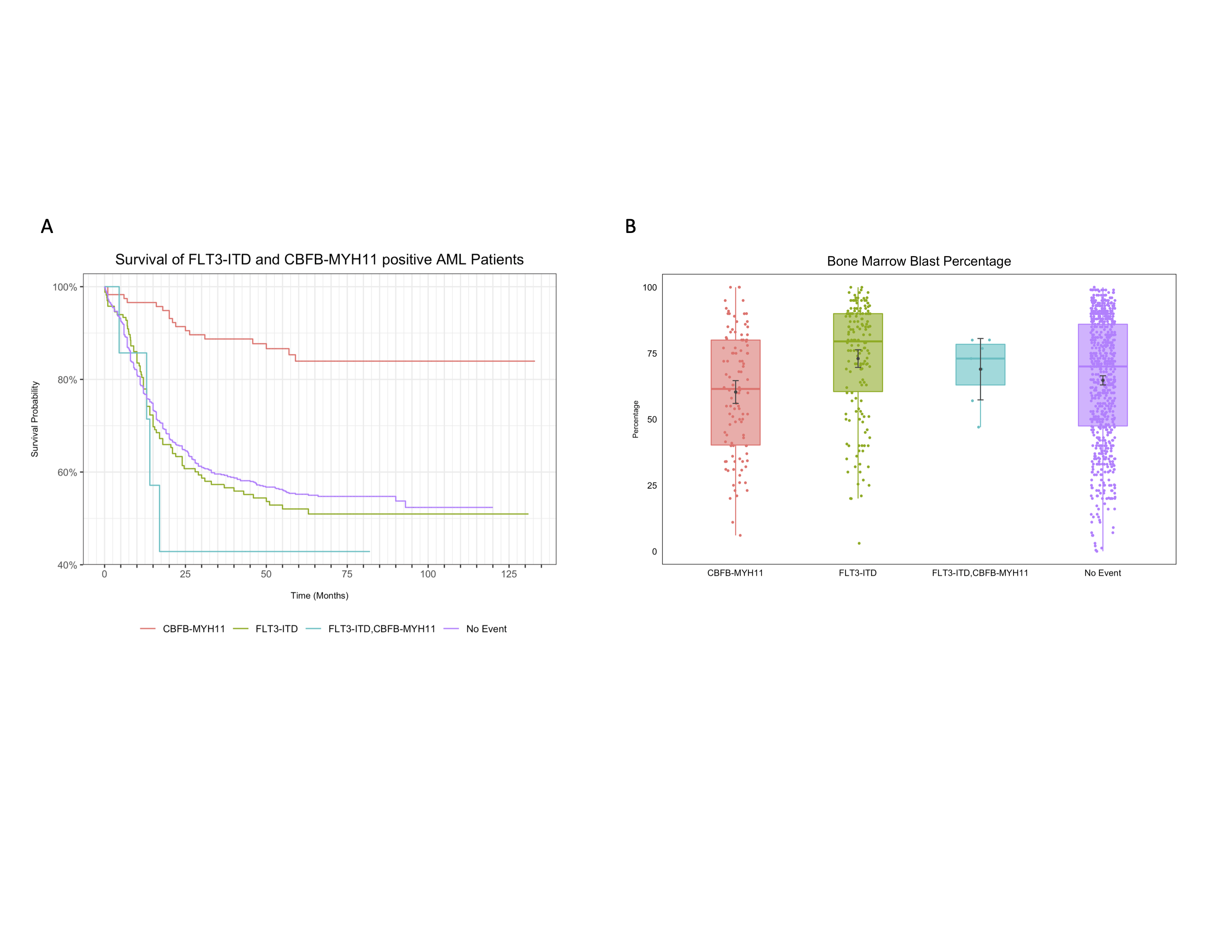

Supplement: Supplementary file 1 — Additional file 1: Figure S1A. Kaplan–Meier curve showing Overall Survival among double mutant, inv16-only, FLT3-ITD only, and double negative cases in TCGA and TARGET studies. B: Blast count distribution in the 4 populations. [file 12967_2022_3486_MOESM1_ESM.tiff]
